# Supplementary material for: Sex- and Co-Mutation-Dependent Prognosis in Patients with SMARCA4-Mutated Malignancies
Source: Cancers (Basel). 2023 May 9;15(10):2665. doi: 10.3390/cancers15102665 (PMC10216441; doi:10.3390/cancers15102665)

Supplemental Figure 1. Kaplan-Meyer curves of overall survival of 13 female and 21 male patients with SMARCA4 mutation/variant with advanced stage cancer identified from The Cancer Genome Atlas (TCGA) dataset.

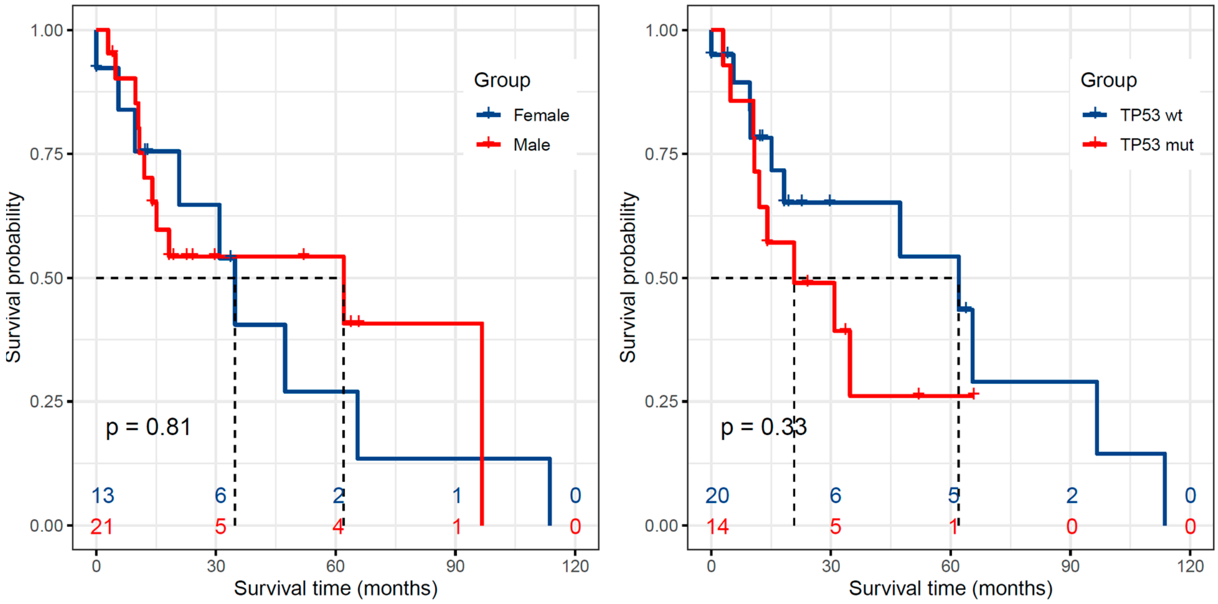

Supplement: Supplementary file 1 [file cancers-15-02665-s001.zip › cancers-2299205-supplementary.pdf]
